# Duplicate Radial Artery Variation: A Comprehensive Analysis With Case Report, Literature Review, Embryological Insights and Clinical Significance

**DOI:** 10.7759/cureus.56976

**Published:** 2024-03-26

**Authors:** Ovanes A Muradyan, Nina I Yotova

**Affiliations:** 1 Anatomy Department, Medical University of Plovdiv, Plovdiv, BGR

**Keywords:** arterial variations in surgery, angiogenesis of upper limb, embryology of upper limb, arterial variations, duplicate radial artery, brachioradial artery, radial artery variation

## Abstract

Thorough knowledge of the anatomical variations of the arterial pattern of the upper limb is of high clinical importance in many medical fields, from surgery to nursery and anesthesiologic practice. During a routine dissection at the Anatomy Department of the Medical University of Plovdiv, a rare variation of the vascular system in the upper limb of a study cadaver was observed. The exhibited variation was the occurrence of a brachioradial artery (BRA) that ran along the main axis of the arm, superficially to the median nerve. After dissection of the cubital fossa, an unusually underdeveloped radial artery was also spotted. Per our knowledge, such a type of duplicate radial artery, the coexistence of a BRA and an underdeveloped radial artery has not been reported in the relevant literature on the topic. The underdeveloped radial artery gave a major branch, the recurrent radial artery, a branch usually given by the brachial artery or the BRA in case of a BRA variation. Variations in the arterial pattern could potentially give insight into the features of the embryological development of the vascular system.

## Introduction

The brachial artery and its two final split branches, the radial and ulnar arteries, provide the main blood supply for the upper limb. It is not uncommon for them to exhibit variations from their usual course. The rarest of the radial artery variations is the duplication of the radial artery with an incidence rate of 0.02% as reported by Rodríguez-Niedenführ et al. (2001 and 2003) [1,2].

The following report explores a case of a duplicate radial artery, a brachioradial artery (BRA), that follows the course of the usual radial artery in the distal forearm region, coexisting with an underdeveloped radial artery with a relatively short length in the proximal forearm region. The two vessels do not anastomose and provide blood supply to different territories of the antebrachial region.

The clinical importance behind the variations of the brachial and the radial artery arises during arterial cannulation and insertion of peripherally inserted central catheters (PICCs) when a deviant artery may be mistaken for a vein [3,4]. Furthermore, such variations may cause an entrapment neuropathy of the median nerve [5]. The presence of a BRA may also be of interest when looking for a source of tissue for coronary artery bypass grafting (CABG) [6]. The various deviations from the typical arterial pattern of the upper limb may contribute to a better understanding of its embryological development.

## Case presentation

**Figure 1:**
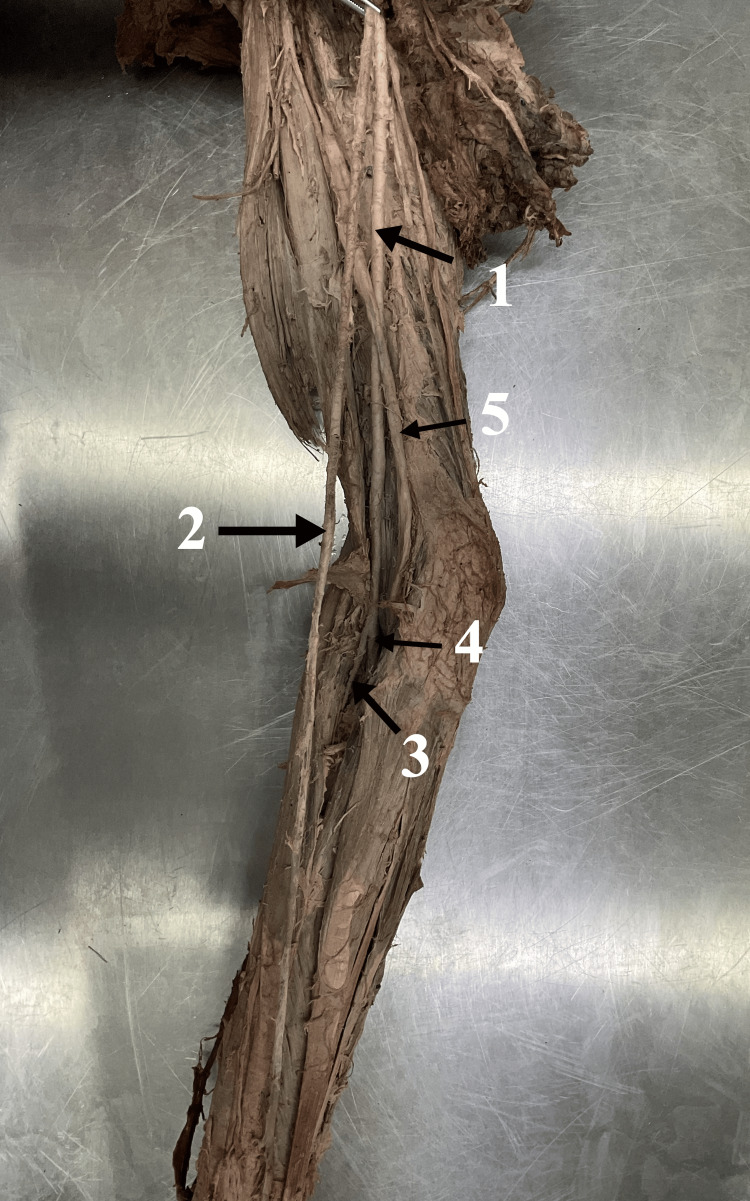
The examined cadaveric right arm image 1. Brachial artery, 2. Brachioradial artery, 3. Radial artery, 4. Ulnar artery, and 5. Median nerve.

During a routine dissection at the Anatomy Department of the Medical University of Plovdiv, a rare variation of the brachial artery (BA) and the radial artery (RA) was observed. The finding was made in the right upper limb of an adult female, preserved in a formaldehyde solution. The axillary artery followed its common course and then continued in the BA. Just 2.7 cm after giving the deep brachial artery (DBA) the BA gave an unusual branch descending down the arm up to the hand. The unusual vessel was positioned superficially to the median nerve (MN), whereas the BA itself continued its common path below it. After a further dissection of the cubital fossa and the carpal region, the artery was classified as a brachioradial artery (BRA). The radial artery also exhibited an anomaly with a greatly reduced length and diameter. No anastomosis between the latter underdeveloped radial artery and the BRA was observed (Figure 1). The ulnar artery (UA) did not exhibit any major variations. The diameter of the BA at different levels is given in the table (Table 1).

**Table 1:** Diameters of the brachial artery in different regions DBA: Deep brachial artery, BRA: Brachioradial artery, RA: Radial artery, UA: Ulnar Artery

Region	Diameter of the brachial artery
Before DBA	5.5 mm
After DBA and before BRA	5 mm
After BRA	4.5 mm
Before the bifurcation of the BA into RA and UA	4 mm

The course of the radial artery (RA)

**Figure 2:**
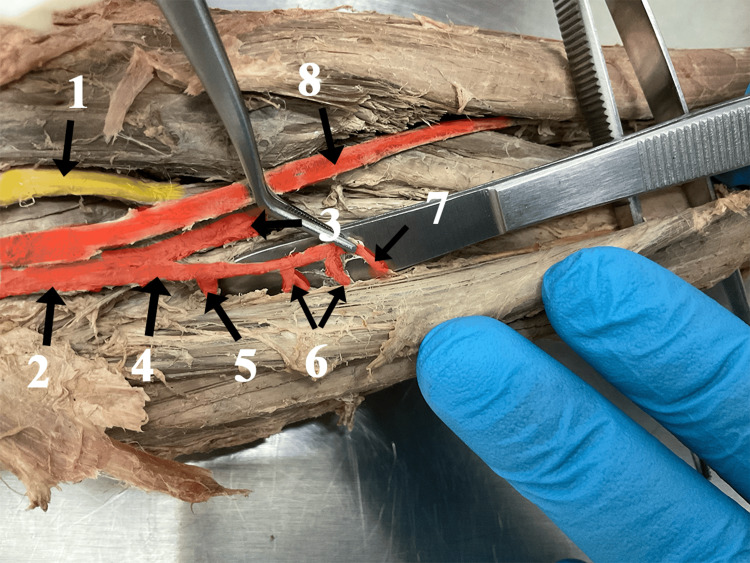
The cadaveric right forearm image with numeric labels 1. Median nerve, 2.Brachial artery, 3.Ulnar artery, 4. Radial artery, 5. Recurrent radial artery, 6. Muscle branches of the radial artery, 7. The final segment of the radial artery, 8. Brachioradial artery.

Starting with a diameter of 2 mm the RA gave the recurrent radial artery (RRA) 1.35 cm after its outset. Then it also gave two branches to the surrounding muscles. With a diameter smaller than usual and a length of 5 cm, the RA finished its course in the extensor carpi radialis brevis muscle (Figure 2).

The course of the brachioradial artery (BRA)

**Figure 3:**
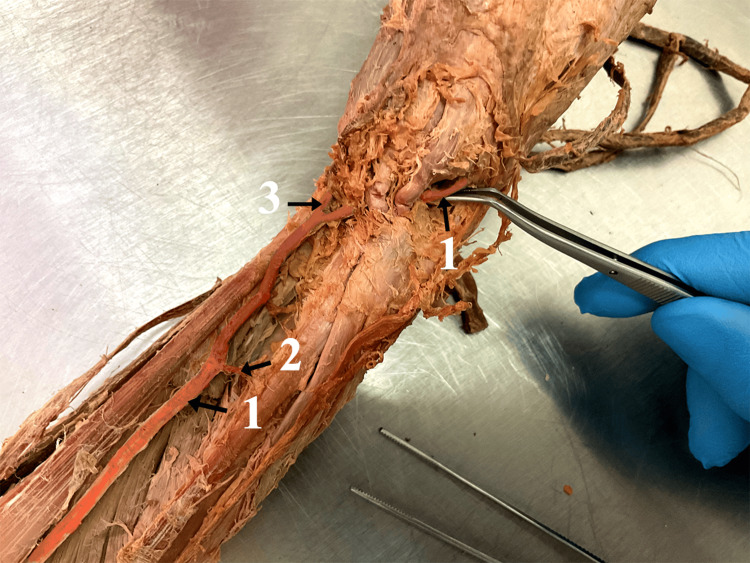
The highlighted brachioradial artery 1. Brachioradial artery (BRA), 2. Muscle branch of BRA, 3. Branch that splits into the ramus carpalis palmaris and ramus palmaris superficialis.

The BRA branched off the BA 2.7 cm after the branching of the DBA. It was positioned along the vertical axis of the anterior brachial region, crossing the MN superficially. Then it passed through the cubital fossa and continued its path in the anterior forearm region. Positioning itself superficially to the underdeveloped radial artery, it took the usual course of the radial artery in the distal forearm region. There, it gave two thinner muscle branches and a wider branch, heading medially which split into the ramus carpalis palmaris and ramus superficialis palmaris. The BRA entered the snuffbox where it gave the ramus carpalis dorsalis and then passed through the interosseus space between the first and second metacarpal bone (Figure 3). The length of BRA, measured from its origin to the branch, which gives r. carpalis palmaris et superficialis palmaris, was 42.5 cm. Its diameter in the different regions is shown in the table (Table 2). The aforementioned arteries were accompanied by the corresponding veins. No nerve variations were observed.

**Table 2:** Diameters of the brachioradial artery

Region	Diameter of the brachioradial artery
At its origin	3.5 mm
At the bicondylar line	3.5 mm
After muscle branches in the carpal region	2.8 mm
After ramus carpi dorsalis	2 mm

## Discussion

The blood supply of the upper limb is typically provided by the axillary artery and its continuation in the brachial artery that eventually splits into the radial and the ulnar artery. Nevertheless, variations of the vascular system may be present. One of the main challenges during the research process was the proper classification of the observed variation. The brachioradial artery variation has an incidence rate of 14%. Its presence together with a radial artery is called a duplication of the radial artery. Rodríguez-Niedenführ et al. studies (2001 and 2003) define the duplication of the radial artery as the rarest of the radial artery variations with an incidence rate of 0.02% [1,2]. They outline it as the coexistence of the superficial brachioradial artery (SBRA) with a normal RA, referring to an article by Kadanoff and Balkansky [7]. According to Salem and Srivastava [8] a duplication of the radial artery is also present when two radial arteries supply different territories. Per our knowledge, the variation described in this report - a duplication of the radial artery caused by a brachioradial artery, coexisting with an underdeveloped radial artery, giving a major branch, the recurrent radial artery, has not been reported in either of the relevant studies.

**Figure 4:**
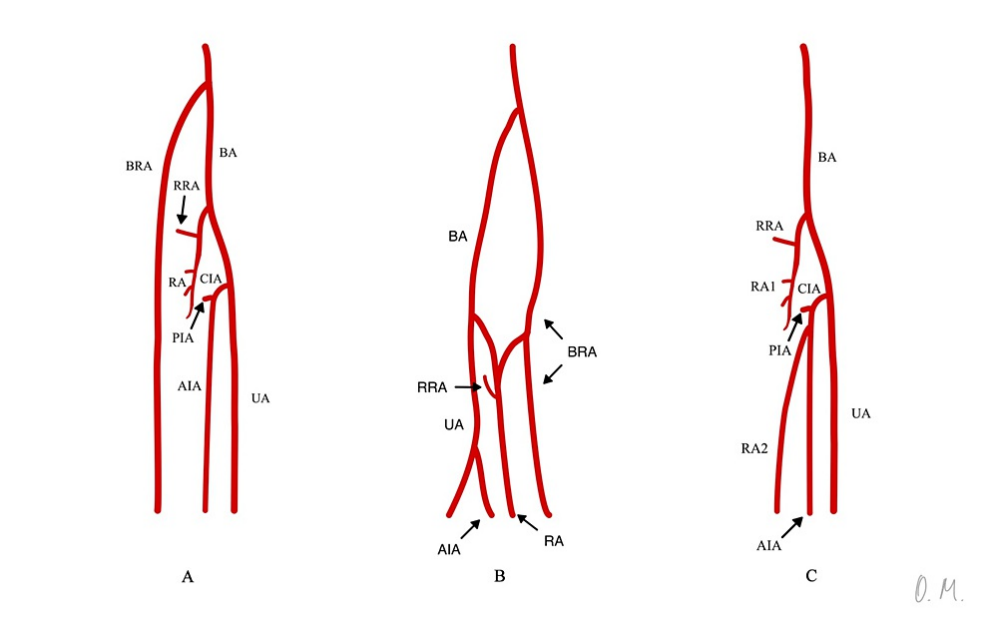
The different types of duplicate radial artery A. The variation represented in this report. Duplication of the radial artery with the coexistence of a BRA and an underdeveloped radial artery; B. The duplicate radial artery variation described by Kadanoff and Balkansky [7]; C. The duplicate radial artery variation described by Sankott AM [12]. BA: Brachial artery, BRA: Brachioradial artery, RA: Radial artery, UA: Ulnar artery, RRA: Radial Recurrent Artery, CIA: Common interosseus artery, AIA: Anterior interosseus artery, PIA: Posterior interosseus artery Image Credit: Ovanes A. Muradyan

In the literature on this topic, only a few cases of duplication of the radial artery have been reported. In some of them, the radial artery splits into two major branches in the forearm region, which may anastomose after a certain distance. Such cases have been reported by Salem et al. [8], Bumbasirević et al. [9], Bhatt et al. [10], and Mohanty et al. [11]. There are only two other reported instances where the duplicated radial artery is not a result of a splitting of the RA itself (Figure 4). The first one is a case reported by Kadanoff and Balkansky [7] involving a coexistence of a superficial brachioradial artery and a normal radial artery. They anastomose in the cubital fossa and then the SBRA continues separately, going over the tendons of the snuffbox and anastomosing with the ramus carpalis dorsalis of the normal radial artery. The other case involves a coexistence of an underdeveloped radial artery and a branch stemming from the anterior interosseus artery. The first behaved as the proximal part of the radial artery, giving the recurrent radial artery, and the latter continued distally in the forearm and hand, taking the distal course of a normal radial artery [12,13].

The variations of the upper limb arteries though rare are of considerable clinical importance for both medical practitioners and nursing professionals. Procedures such as intravenous injections and insertions of a peripheral intravenous cannula (IV cannula) or a PICC are regularly performed during medical emergencies, surgeries, chemotherapy, or prolonged hospitalization. After spotting the pulse and the location of the brachial artery, the medical professional might confuse the brachioradial artery for a vein and attempt to perform the listed procedures on it. This may cause severe bleeding in the area and lead to further complications. Such cases of unintentional injections due to similar anomalies of the arterial system in the upper limb have been reported by Shivappagoudar and George [3] and Rai et al. [4]. The occurrence of an atypical arterial pattern might be a risk factor for an iatrogenic injury during surgical operations in the cubital and brachial region, especially in the case of a fracture of the humerus that requires surgical intervention.

The deviant morphological pattern of the blood vessels in the instance of a brachioradial artery may be a cause for median nerve compression neuropathy. This is due to the fact that the nerve passes between the brachial and the brachioradial artery. Such a condition might cause both palsy and loss of sensation in parts of the affected limb. A case of median nerve neuropathy caused by a superficial brachial artery has been reported by Liu et al. [5].

The presence of the brachioradial artery in patients could potentially be of medical importance during coronary artery bypass grafting (CABG). The radial artery is considered the second option as a conduit (radial artery graft) after the left internal mammary artery (left internal mammary graft) in CABG. Nappi et al. [6] explore the use of the radial artery for this purpose and the possible anatomical variations of the radial artery such as high origin. They conclude that the existence of such an anomaly usually does not prevent the use of the artery as a conduit but rather requires slight changes in the operative technique.

The vascular supply of the upper limb develops alongside the development of the limb itself. The process of angiogenesis in the upper limb starts with the formation of a primitive capillary plexus at the base of the limb bud and a terminal capillary plexus at the tip of the limb bud. Bilaterally, the seventh cervical intersegmental artery gives a lateral branch to the upper limb that later becomes the axial artery. The axial artery develops into the axillary artery, the brachial artery, and the anterior interosseus artery that connects to the terminal capillary plexus. The superficial brachial artery is usually a temporary embryological vessel that plays an important role in the morphogenesis of the upper limb. It has two final branches: a medium one, which is involved in the development of the median and the ulnar artery, and a collateral one that gives the radial artery. The medium one branches into a median and an ulnar branch that anastomose with similar branches from the brachial artery and then subverts thus forming the median and the ulnar artery. Then the collateral branch, which is de facto the continuation of the superficial brachial artery, anastomoses with a deep branch of the brachial artery that later on dominates hemodynamically and becomes the radial artery. As a result, the proximal part of the superficial brachial artery that is located before the anastomosis regresses [14].

The occurrence of a brachioradial artery and an underdeveloped radial artery might be caused by an external interference that disturbed the formation of an anastomosis between the collateral branch of the superficial brachial artery and the radial branch, originating from the brachial artery. The first one might have developed into the brachioradial artery, which takes part in the blood supply of the distal part of the upper limb, whereas the second one might have stayed as a shorter vessel (underdeveloped radial artery) that gave the recurrent radial artery and muscles branches to the deeper muscles of the forearm.

## Conclusions

Extensive anatomical knowledge of the arterial system of the upper limb and its variations is required in many fields of medicine such as traumatological, cardiovascular and oncological surgery, radiodiagnostics, anesthetic and nursing practice. The duplication of the radial artery, when caused by the existence of an additional brachioradial artery, may be a risk factor during insertion of IV cannulas or peripherally inserted central catheters (PICCs). Its potential presence should also be taken into consideration during surgical interventions in the area such as those due to fractures of the surrounding bones. The brachioradial artery could also be used as a conduit for CABG. Arterial variations might also serve as a different viewpoint in understanding the embryological development of the arterial pattern of the upper limb.
